# A Consensus Proposal for Nutritional Indicators to Assess the Sustainability of a Healthy Diet: The Mediterranean Diet as a Case Study

**DOI:** 10.3389/fnut.2016.00037

**Published:** 2016-08-29

**Authors:** Lorenzo M. Donini, Sandro Dernini, Denis Lairon, Lluis Serra-Majem, Marie-Josèphe Amiot, Valeria del Balzo, Anna-Maria Giusti, Barbara Burlingame, Rekia Belahsen, Giuseppe Maiani, Angela Polito, Aida Turrini, Federica Intorre, Antonia Trichopoulou, Elliot M. Berry

**Affiliations:** ^1^Sapienza University of Rome, Rome, Italy; ^2^CIISCAM-International Inter-University Center for Mediterranean Food Culture Studies, Rome, Italy; ^3^Food and Agriculture Organization of the United Nations, Rome, Italy; ^4^INRA 1260, INSERM 1062, Aix Marseille University, Marseille, France; ^5^CIBER OBN, Instituto de Salud Carlos III, University of Las Palmas de Gran Canaria, Las Palmas, Spain; ^6^Deakin University, Melbourne, Australia; ^7^Chouaib Doukkali University, El Jadida, Morocco; ^8^Council for Agricultural Research and Economics-Research Center on Food and Nutrition (CRA-NUT, formerly INRAN), Rome, Italy; ^9^Hellenic Health Foundation, Athens, Greece; ^10^Braun School of Public Health, Hebrew University-Hadassah Medical School, Jerusalem, Israel

**Keywords:** sustainable diets, nutrition indicators, Mediterranean diet, dietary energy density, dietary nutrient density, dietary diversity, physical activity, non-communicable chronic diseases

## Abstract

**Background:**

There is increasing evidence of the multiple effects of diets on public health nutrition, society, and environment. Sustainability and food security are closely interrelated. The traditional Mediterranean Diet (MD) is recognized as a healthier dietary pattern with a lower environmental impact. As a case study, the MD may guide innovative inter-sectorial efforts to counteract the degradation of ecosystems, loss of biodiversity, and homogeneity of diets due to globalization through the improvement of sustainable healthy dietary patterns. This consensus position paper defines a suite of the most appropriate nutrition and health indicators for assessing the sustainability of diets based on the MD.

**Methods:**

In 2011, an informal International Working Group from different national and international institutions was convened. Through online and face-to-face brainstorming meetings over 4 years, a set of nutrition and health indicators for sustainability was identified and refined.

**Results:**

Thirteen nutrition indicators of sustainability relating were identified in five areas. *Biochemical characteristics of food* (A1. Vegetable/animal protein consumption ratios; A2. Average dietary energy adequacy; A3. Dietary Energy Density Score; A4. Nutrient density of diet), *Food Quality* (A5. Fruit and vegetable consumption/intakes; A6. Dietary Diversity Score), *Environment* (A7. Food biodiversity composition and consumption; A8. Rate of Local/regional foods and seasonality; A9. Rate of eco-friendly food production and/or consumption), *Lifestyle* (A10. Physical activity/physical inactivity prevalence; A11. Adherence to the Mediterranean dietary pattern), C*linical Aspects* (A12. Diet-related morbidity/mortality statistics; A13. Nutritional Anthropometry). A standardized set of information was provided for each indicator: definition, methodology, background, data sources, limitations of the indicator, and references.

**Conclusion:**

The selection and analysis of these indicators has been performed (where possible) with specific reference to the MD. Sustainability of food systems is an urgent priority for governments and international organizations to address the serious socioeconomic and environmental implications of short-sighted and short-term practices for agricultural land and rural communities. These proposed nutrition indicators will be a useful methodological framework for designing health, education, and agricultural policies in order, not only to conserve the traditional diets of the Mediterranean area as a common cultural heritage and lifestyle but also to enhance the sustainability of diets in general.

## Introduction

There is increasing evidence of the multiple effects and cost of diets on public health nutrition, society, and environment (1–4). Sustainability and food security are closely interrelated (5).

Food systems around the world are changing rapidly, with profound implications for diets and nutritional outcomes. The sustainable diets concept (6) highlights the role of sustainable consumption as a driver of sustainable production, for food systems’ transformation toward more sustainable food consumption and production patterns, which are among the most important drivers of environmental pressures (7, 8). Food systems need to grow within the framework of finite and often reduced funds and need to make use of natural resources and skills in a sustainable manner to conserve the fragile ecosystem balance. There is an increasing need to develop a holistic view on sustainable food systems, from production to consumption and diets. This can be achieved through linkage to the enhancement of more sustainable dietary models. In the early 1980s, the notion of “sustainable diets” arose to recommend diets, which would be healthier for both the environment as well as for consumers (9). With food globalization and the increased industrialization of agricultural systems, the concepts of sustainable diets and agro-food systems had been neglected. In the last decade, the interest in sustainable diets has been revived by a growing body of scientific evidence of the non-sustainability of current dietary trends (10–13). However, it is not clear that high nutritional quality is always associated with low environmental impact (14).

The traditional Mediterranean Diet (MD) has been studied in-depth and recognized as a healthier dietary pattern characterized by a lower environmental impact (15–18).

The health benefits of the MD in preventing chronic diseases have been well recognized by the scientific community, since the pioneer Seven Countries Study, conducted by Ancel Keys, established the association of a traditional Mediterranean dietary pattern with markedly reduced coronary heart disease mortality (19–21). Later, additional benefits of the MD have been widely reported scientifically for diseases other than cardiovascular, such as obesity, diabetes, cancer, depression, cognitive decline as well as improved quality of life (22–30).

But, despite these well-documented health benefits and the low environmental impact of the MD, current surveys show a decline in its adherence in Northern, Southern, and Eastern Mediterranean countries, because of multifactorial influences – lifestyle changes, globalization of food markets, and economic and sociocultural factors (26, 31–39).

The three main domains of sustainability – economic, social, and environmental – need to be integrated into the dimensions of nutrition, health, and culture. During several recent international seminars, four main thematic areas of sustainability have been identified (1) nutrition, health, and lifestyle; (2) environment including agro-biodiversity; (3) economy; (4) society and culture (40). The assessment and development of sustainable diet models requires awareness among consumers, producers, and governments that agriculture, food, nutrition, health, culture, environment, and sustainability are strongly interdependent.

The MD, as a case study, may guide innovative inter-sectorial efforts to counteract the degradation of ecosystems, loss of biodiversity, and homogenization of diets due to globalization through the improvement of sustainable dietary patterns with their health benefits. For this purpose, it is necessary to define the sustainability of diets through the analysis of evidence, development of methods and indicators, and the development/promotion of policy guidelines.

In particular, in the context of sustainable consumption and production, indicators are necessary to monitor time-trends whether a society’s consumption and production patterns lead to more socially equitable and environmentally sustainable development. They are also necessary to evaluate the impact of dietary patterns on long-term health status and, in particular, on the pathogenesis and incidence of non-communicable chronic diseases. A number of international organizations, as well as different governments, have developed sets of indicators for sustainable consumption and production, mostly as attempts to monitor sustainable development, but also as part, or in support, of dedicated sustainable consumption and production strategies (41).

However, from a methodological approach, there are at least three diverging criteria to define sustainability indicators. (1) According to the International Institute for Sustainable Development “an indicator quantifies and simplifies phenomena and helps us understand complex realities” (42). (2) According to the Organization for Economic Cooperation and Development, an indicator is “a parameter, or a value derived from parameters, which points to, provides information about, or describes the state of a phenomenon/environment/area, with a significance extending beyond that directly associated with its value” (43). (3) According to Food and Agriculture Organization of the United Nations (FAO), an indicator does not reduce to the data on which it is based; it generally comprises elements (a cutoff value, a frame of reference, a mode of expression, etc.), which allow a relatively universal appreciation of the information it supplies and also facilitate comparison in time and space (44–46).

This consensus position paper attempts to define a non-exhaustive ensemble or suite of the most appropriate nutrition and health indicators for assessing the sustainability of diets, using the MD as a case study.

## Methods

An International Working Group was informally developed in 2011 with the contribution of different national and international institutions – FAO; Sapienza University of Rome, Italy; University of Marseille, France; Hebrew University of Jerusalem, Israel; University of Las Palmas, Spain; Chouaib Doukkali University, El Jadida, Morocco; Centre International de Hautes Etudes Agronomiques Méditerranéennes (CIHEAM); International Inter-University Center for MEDITERRANEAN Food Culture Studies (CIISCAM); Council for Agricultural Research and Economics-Research Center on Food and Nutrition (formerly INRAN) (CRA-NUT), Rome, Italy; International Foundation of Mediterranean Diet (IFMED); Hellenic Health Foundation – Greece; and Forum on Mediterranean Food Cultures. Its purpose was to define the nutritional and health indicators relevant to assessing the sustainability of the diets, and in particular, of the MD.

Through online and face-to-face brainstorming meetings, held from November 2011 to April 2015, a set of nutrition and health indicators of sustainability was identified. The definitions and the characteristics of the nutrition and health indicators were progressively refined based on the recursive comments of the participants (Table 1).

**Table 1:** **Milestones for the definition of nutritional indicators of sustainability of Mediterranean Diet**.

CIHEAM MAI–Bari International Workshop on “Guidelines for Improving the Sustainability of the Mediterranean Diet,” Bari, November 28–29, 2011 FAO/CIHEAM discussion paper on “Towards the Development of Guidelines for Improving the Sustainability of Diets and Food Consumption Patterns in the Mediterranean Area” (40, 45) CIHEAM/FAO Seminar on “Food Systems and Sustainable Diets: The Mediterranean Diet as a Pilot Study” in preparation of the ninth Meeting of CIHEAM Ministers of Agriculture, Malta, September 25–26, 2012 (46) CRA/FQH International Workshop on “Assessing Sustainable Diets within the Sustainability of Food Systems,” Rome, September 15, 2014; CIISCAM/Sapienza University of Rome, fourth Carlo Cannella Meeting, Rome, February 26, 2015.

The process for the production of the present document began in the second half of 2014. The manuscript draft was then circulated among the participants of the different institutions. An agreement on the final version of the document was reached in April 2015 in Rome.

Different instruments were used to have a comprehensive picture of the eating patterns (Table 2). Individual Dietary Surveys (IDS), Household Budget Surveys (HBS), and food balance sheets (FBS) vary in the methodology that leads to different levels of disaggregation and detail. Usually, for one food item (after alignment), the mathematical relation in relation to quantities is FBS > HBS > IDS, because FBS items are calculated excluding reuse and stock variation (national account budgets); they represent what food items are *available* per capita, but not obviously what is necessarily *consumed*. HBS do not include meals eaten outside the home but does include kitchen wastes and leftovers. IDS refer to edible part (excluding wastes and leftovers) of food and include eating at home and outside of home (47). Finally FBS overestimated food consumption and nutrient intake compared to IDS. Results between HBS and IDS are quite similar, except for fish, meat, pulses, and vegetables, which are underestimated by HBS, and sugar, honey, and cereals, which are overestimated.

**Table 2:** **Data sources and criteria used for the definition of nutritional indicators of Mediterranean Diet**.

– FBS and commodity balances provide data for domestic availability of a food, or food component in the case of protein. The contributing data include the sum of production and imports, with exports and non-food use subtracted. New modules to the FAOSTAT family of databases, including land use, emission, pesticide, fertilizer, and irrigation, will provide more data on environmental sustainability when analyzed with protein ratio data. Food consumption studies, national nutrition surveys, household budget surveys, etc., will be available in some countries to provide accurate individual data instead of FAO FBS data. It is noted that FBS data represent what food is *available* per capita on a national scale, but not what is actually eaten. – HBS are national surveys mainly focusing on consumption expenditure. They are conducted in all EU Member States and their primary aim (especially at national level) is to calculate weights for the consumer price index. They were launched in most EU Member States at the beginning of the 1960s, and Eurostat has been collating and publishing these survey data every 5 years since 1988. The two last collection rounds were 2005 and 2010. Although there have been continuous efforts toward harmonization, differences remain. – IDS is a class of methodologies including methods with various precision level (food record, 24-h recall, Food Frequency Questionnaire, dietary history, food propensity questionnaires, and combinations) usually not carried out at regular interval time except for some national reality as, e.g., the NHANES program in the USA (http://www.cdc.gov/nchs/nhanes.htm) or the NDNS (https://www.gov.uk/government/uploads/system/uploads/attachment_data/file/310997/NDNS_Y1_to_4_UK_report_Executive_summary.pdf) in the UK. The European Food Safety Authority has launched the EU Menu (http://www.efsa.europa.eu/en/datexfoodcdb/datexeumenu.htm) program to push Member States of the European Union to harmonize dietary surveys. FBS and HBS for Europe are the source complying with the definition of indicators (see “Criteria for Selecting Indicators”).

The worldwide data FBS^1^ and the European data HBS^2^ are current statistics. An overall view of HBS with the aim of harmonizing data codes for nutritional analysis can be found in the publication related to the DAFNE project^3^ (48), and the results are also published in the “European Nutrition and Health Report 2009”^4^ (49).

Important information on Agri-environmental indicators (AEIs), HBS, and from farm to fork statistics may also be collected from Eurostat.^5^

## Criteria for Selecting Indicators

To select the most effective indicators, the following criteria were considered (41):

1. *Relevant to the question being asked*. The indicator should be the best indicator currently available to answer the question;
2. *Understandable*, i.e., clear, simple, and unambiguous;
3. *Graphically representable*;
4. *Readily interpretable*, i.e., clear, which direction the indicator should develop to lead to greater sustainability;
5. *Relevant* in most Economic European Area Member and collaborating countries, i.e., not restricted to an issue, which is limited to a few member countries;
6. *Monitorable*, i.e., based on data that are readily available in member and collaborating countries, or could be made available at reasonable cost–benefit ratio and with regularity within time frame of policy cycle (i.e., updated each year and with maximum 4-year time delay);
7. *Reliable and consistent*, i.e., data collection and analysis methodologies should preferably be consistent from country to country, and at very least, be consistent within a given country from year to year;
8. *Representative*, i.e., can be taken to represent current sustainable consumption and production trends within a given sector, final consumption cluster, etc.

## Results

A set of nutrition indicators of sustainability was identified by the Working Group, and thirteen indicators, from A1 to A13, were finalized.

**Table:** 

Biochemical characteristics of food	A1. Vegetable/animal protein consumption ratios
	A2. Average dietary energy adequacy
	A3. Dietary Energy Density Score
	A4. Nutrient density of diet
Food quality	A5. Fruit and vegetable consumption/intakes
	A6. Dietary Diversity Score
Environment	A7. Food biodiversity composition and consumption
	A8. Rate of local/regional foods and seasonality
	A9. Rate of eco-friendly food production and/or consumption
Lifestyle	A10. Physical activity/physical inactivity prevalence
	A11. Adherence to the Mediterranean dietary pattern
Clinical aspects	A12. Diet-related morbidity/mortality statistics
	A13. Nutritional anthropometry

For each indicator, the following set of information is provided: definition, methodology, background, data sources, limitations of the indicator, and references.

### A1. Plant and Animal Protein Consumption Ratios

#### Definition

This indicator is a ratio of the relative intakes of protein from plant and animal sources, assessing adherence to an optimal dietary pattern, and a proxy for environmental impact of diets.

#### Methodology

Parameter considered is as follows:

– ratio of plant (cereals, vegetables, pulses, fruit) and animal (meat, fish, eggs, dairy products) proteins in the diet using existing data.

Adherence to an optimal ratio, including the MD, can be judged by simple comparison, and the trend can be monitored over the time series of available data, regardless of the data source.

#### Data Sources

FAOSTAT FBS and commodity balances provide data for domestic availability of a food, and food component in the case of protein (50). The contributing data include the sum of production and imports, with exports and non-food use subtracted. New modules to the FAOSTAT family of databases, including land use, emission, pesticide, fertilizer, and irrigation, will provide more data on environmental sustainability when analyzed with protein ratio data. Food consumption studies, National Nutrition Surveys, HBS, etc., will be available in some countries to augment or replace FAOSTAT data.

#### Limitations of the Indicator

FAOSTAT data from FBS and commodity balances reflect domestic availability of foods, not consumption or production *per se*. While these data have proven useful for assessing nutritional adequacy of diets, with a long history of use, they may significantly misrepresent sustainability issues. For example, livestock production has a greater role in GHG emission than livestock consumption. If meat is imported rather than domestically produced, the calculation of environmental impact may be skewed if using food balance or commodity balance datasets. Similarly, National Nutrition Surveys do not address the issue of production. Food losses and waste not accounted for in the datasets will affect the calculations and interpretation. Additionally, the advantages of using plant:animal protein ratio, as opposed to plant:animal dietary energy ratio or plant:animal ratio in grams per person per day, need to be elaborated (51, 52).

### A2. Average Dietary Energy Adequacy

#### Definition

The indicator expresses the dietary energy supply (DES) as a percentage of the average dietary energy requirement (ADER) in the country. Each country’s or region’s average supply of calories for food consumption is normalized by the ADER estimated for its population, to provide an index of adequacy of the food supply in terms of calories. This indicator was proposed by FAO as new approach for the measurement of food security (53).

#### Methodology

Parameters considered are as follows:

– dietary energy supply (kilocalories/capita/day): average supply available for each individual in the total population (it does not indicate what is actually *consumed* by individuals);
– average dietary energy requirement (kilocalories/capita/day): the amount of food energy needed to balance energy expenditure in order to maintain body size, body composition, and a level of necessary and desirable physical activity consistent with long-term good health.

#### Data Sources

Data can be downloaded from http://www.fao.org/economic/ess/ess-fs/ess-fadata/en/#.VPhu3y7K1i0. Otherwise, DES can be obtained from National IDS, HBS, FBS, and ADER from National Energy Requirements, FAO Human Energy Requirements.

#### Limitations of the Indicator

National IDS using food diaries or dietary recalls estimate the actual consumption (i.e., the dietary energy intake), provide the best evidence on food consumption, and constitute the best method for assessing energy intake, and more generally, dietary patterns and evaluating diet–disease associations. Being expensive and labor-intensive, these surveys are undertaken only in a limited number of countries, often at regional or local level or in specific population groups; furthermore, it is difficult to accomplish comparability at the international level, because the assessment methods are variable, self-reported, and consequently subject to considerable measurement errors. In order to overcome these problems, the per capita DES can be used instead of the dietary energy intake. Data on DES can be gathered from FBS and HBS. FBS and HBS overestimate energy intake, although not in a linear way; while FBS includes eating out, HBS does not; food losses and waste should be considered. In order to reduce the impact of possible errors in estimated DES, due to the difficulties in properly accounting of stock variations in major food, the indicator is calculated as an average over 3 years (for example, 2010–2012, 2011–2013, and 2012–2014).

Average dietary energy requirement is a reference for adequate nutrition in the population. The recommended level of dietary energy intake for a population group is the mean energy requirement of the healthy, well-nourished individuals who constitute that group. The estimates of requirements derived from measurements of a collection of individuals of the same age, gender, body size, presumed body composition, and physical activity are grouped to give the average energy requirements for a class of people or a population group. These requirements are used together to predict the requirements of other individuals with similar characteristics, but on whom measurements have not been made. Consequently, application of these results to any one individual for clinical or other purposes may lead to errors of diagnosis and improper management (54). On the other hand, data on the size of consumed food portion that influence energy intake need to be evaluated (55), as positive relationships between portion size and energy intake have been demonstrated in adults (56–58).

### A3. Dietary Energy Density Score

#### Definition

This indicator measures the amount of energy (kcal or kJ) in a given weight (g, 100 g, or kg) of diet as a proxy for healthy dietary patterns.

#### Methodology

Parameter considered is as follows:

– dietary energy density (kilocalories/gram) calculated by dividing total dietary energy by the edible weight of foods and caloric beverages consumed. The primary data are as follows:
  - the mean amounts of various foods/beverages or food groups consumed daily.
  - the energy provided by weight unit of the foods/beverages or food groups as provided by food composition databases. It will be expressed as the amount of energy (kilocalories or kilojoules) in 100 g or 1 k of daily diet.

#### Data Sources

National IDS, HBS, and FAO FBS.

#### Limitations of the Indicator

Individual Dietary Surveys provide the most accurate figures for actual daily food consumption. Data obtained from FBS do not reflect the effective food intake, because they relate to the food quantities theoretically available for consumption; the amount of food consumed is lower than those reported in FBS, due to the degree of losses of edible food and nutrients in the household, e.g., during storage, in preparation, and cooking, as plate waste or quantities fed to domestic animals and pets, or thrown away. Depending on data sources and studies, the level of accuracy and units used can vary.

Also, the data obtained even from National Dietary Surveys do not reflect the portion size. Indeed, there is evidence that larger portion size of energy-dense and nutrient-poor foods is involved in the increase of overweight and obesity that accompany the changes in dietary patterns in children and adults (57, 58).

### A4. Nutrient Density of Diet and Foods

#### Definition

The nutrient density of a composite diet is the amount of various necessary nutrients and fibers present in a given daily diet expressed in weight (or evenly in energy).

#### Methodology

Parameters considered are as follows:

– a daily diet:
  - mean adequacy ratio (MAR) based on the mean percentage of the recommended intakes for 29 key needed nutrients, alone or in combination with the mean excess ratio (MER) for nutrients to limit.
– general purpose:
  - the amount of every nutrient present in a unit of a given food/beverage or food group as provided by food composition databases;
  - Nutrient Density Scores referring to either 100 g, 100 kcal/kJ, or cost/kg or L of a given food: ex simplified SAIN/LIM scoring (Score d’Adéquation Individuel aux Recommandations Nutritionnelles – SAIN; nutrients to be limited – LIM) (10).

Some publications cited in the reference list provide examples of calculations and interpretations (12, 59).

#### Data Sources

National IDS, HBS, and FAO FBS (see indicator A2 for a comprehensive description).

#### Limitations of the Indicator

All scores designed to evaluate the nutrient density of either individual foods or whole diet have advantages and limitations. They must be taken into account depending on the precise context and objective considered. The limitations are (i) the need for accurate and quantitative dietary intake data and food composition databases; (ii) comparisons between countries are limited by possibly different daily recommended intakes (energy, nutrients, and fiber); and (iii) comparisons between studies need the use of the same nutrients and total number of nutrients.

It has to be considered that the MAR normally should be 29, but because of the lack of composition tables, the number is usually less. In France, for example, 23 includes the different lipids.

### A5. Fruit and Vegetable Consumption/Intakes

#### Definition

This indicator is the measure of the consumption (grams/capita/day) of fruits and vegetables, including pulses, nuts, and seeds (12), directly applicable to assessing adherence to MD, and as a proxy for a healthy diet and specific micronutrient intakes.

#### Methodology

Parameter considered is as follows:

– measure of the consumption (supply, availability, intake) of fruits and vegetables (grams/capita/day), including pulses, nuts, and seeds.

#### Data Sources

National IDS, HBS, and FAO FBS.

#### Limitations of the Indicator

Data obtained from FBS do not reflect the effective food intake, because they relate to the food quantities available to the consumer (but not necessarily consumed). Thus, the amount of food consumed is usually lower than those reported in FBS (59), due to the degree of losses of edible food and nutrients in the household/catering, e.g., during storage, in preparation, and cooking, as plate waste or quantities fed to domestic animals and pets, or thrown away. However, when National IDS are not available, the HBS and/or FBS provide good indicators by which to compare several countries and different time periods.

Although it is not specified in official documents, considering the high proportion of waste often present in preparations of plant foods, it should be specified that the weight of 400 g daily refers to edible product net of waste (12, 60).

When using national supply data, the reference value could be increased to take into account that goods include inedible parts. Moreover, 500 g per day are also recommended in some dietary guidelines^6^ and for ischemic heart disease prevention (61, 62).

### A6. Dietary Diversity Score

#### Definition

Dietary diversity is a qualitative measure of the household access and consumption of a wide variety of foods. It is an indicator that reflects the households’ diet quality and is also a proxy for the adequacy of nutrient intake of the diet for individuals (63–65). This concept is based on the fact that the needs in nutrients are not covered by a single food, but by a diet composed of several foods.

It is associated with household socioeconomic status and food security (energy availability at the household level). A greater dietary diversity was also reported to protect different households against the double burden of malnutrition known in countries in nutrition transition (66–68).

#### Methodology

Parameters considered are as follows:

– Dietary Diversity Scores (DDS) defined as the number of food groups consumed over a reference period:
  - Individual Dietary Diversity Score (IDDS) used as proxy of the nutritional quality of individual diet has for aim to assess the adequacy of nutrient intake;
  - Dietary Diversity Score at the household level (HDDS) is used, on the other hand, as proxy of the socioeconomic level of the household and intends to reflect the economic ability of a household to consume a variety of foods.
– Dietary Variety Score (DVS) (69) corresponding to the number of foods consumed among a list of foods.
– US Healthy Food Diversity index (70), a tool for the simultaneous measurement of dietary variety, quality, and proportionality at individual level.

#### Data Sources

Usually, specific questionnaires are administered. The use of National IDS, HBS, and FAO FBS need to be experimented.

#### Limitations of the Indicator

Even if there is a preference for the DDS based on food groups, the issue of the number and the choice of these food groups has not yet been resolved. The selection of food groups can be guided by the objectives for which the scores are used.

For example, if the score of diversity is used to identify populations at risk of micronutrient deficiency, the classification used should distinguish food groups depending on their content in micronutrients. In this case, it is obvious that comparisons between studies or countries are more difficult.

Moreover, it has to be considered that the diversity scores have been designed specifically for developing countries without regularly carried out national statistics about this topic.

### A7. Food Biodiversity Composition and Consumption

#### Definition

Biodiversity covers diversity within species, between species, and of ecosystems; synonyms are biological diversity and ecological diversity. For the purposes of human nutrition, biodiversity refers to foods identified at the taxonomic level below species (e.g., cultivar, breed) or by local varietal name, and wild, neglected, and/or underutilized species. Biodiversity is distinctly different from “dietary diversity,” which reflects intake at the level of aggregate food groups.

#### Methodology

Parameters considered are as follows:

– food composition: a count of the number of foods:
  - at variety/cultivar/breed level for common foods with at least one value for component found in published and unpublished sources.
  - at species level for wild/indigenous/underutilized foods with at least one value for component found in published and unpublished sources.
– food Consumption:
  - the taxonomic diversity of foods, as for food composition, reported in food consumption/dietary intake surveys. Data collected and reported include:
    ▪ the study instrument (e.g., diet history, food frequency) with details (scope, date, number, and description of subjects, geographical/ethnic coverage; reference, total number of studies examined);
    ▪ the qualifying biodiverse foods reported (number of foods, food lists);
  - the number of surveys with at least one reported food counting for biodiversity.

#### Data Sources

Food and Agriculture Organization of the United Nations/International Network of Food Data Systems (INFOODS) compile data and report periodically (71). For food composition, data are obtained by searching peer-reviewed journals using the search engines Scopus and Science Direct, and through a call for data conducted *via* INFOODS. These data are then compiled in a Biodiversity Food Composition Database (72).

For food consumption, data are obtained from all surveys, including National Nutrition Surveys, market surveys, ethnobiological investigations, and inventory studies. All published and unpublished available resources are searched, including peer-reviewed journals, official international/regional/national/subnational survey reports, conference presentations, and published matter, including posters, abstracts published from meetings, and theses.

#### Limitations of the Indicators

The development and reporting on the indicators are recent, and only two to three time points are available. The usefulness of the indicators should be assessed in the future and judged against market survey data as well as nutritional outcomes. For the moment, the results represent a reflection of the attention being paid to biodiversity by researchers designing food composition studies and dietary surveys. Monitoring and reporting on the biodiversity indicators is the responsibility of FAO/INFOODS. It is a time-consuming activity, and, for the 2014–2015 biennium, FAO has put few or no resources into the continuation of this effort.

### A8. Local/Regional Foods and Seasonality

#### Definition

The term “local food system” (or “regional food system”) is used to describe a method of food production and distribution that is geographically localized, rather than national and/or international. Food is grown (or raised) and harvested close to consumers’ homes, then distributed over much shorter distances than is usual in the conventional globalized industrial food system with long-distance transportation. In general, local/regional food systems are associated with the sustainable agriculture concept, but not systematically. In particular, it is based on purchases at short distance from the producer (from few to 100 km or miles) and directly from the producer or with one intermediate between the consumer and producer.

Production “in season” means that minimum artificial conditions are used to grow the products (essentially plant products: vegetables and fruits), without heated greenhouses in the local agro-environmental conditions and no long-term cold storage).

#### Methodology

Parameters considered are as follows:

– the distance between consumer purchase location and producing area; it is usually considered that it should be at maximum 150 km (around 100 miles).
– the number of intermediates between producer and consumer with zero when direct from producer, one when one intermediate is present (one can be considered as a cut point for discrimination).
– the consumer choice:
  - directly to local/regional producers (on-farm, farmer’s market/shop, food baskets made of local foods) as a share of total food purchases,
  - share of fresh vegetables or fruits consumed coming from open field or unheated greenhouse cultivation.
– the duration between fruit harvest (known or estimated from agriculture statistics of the concerned growing location or country) and purchase of fresh fruit, as a direct reflect of distance from seasonal production (and cold storage duration).

#### Data Sources

The information necessary to assess these indicators can be only obtained from dedicated studies where such specific questions are addressed. It is the case in some national human cohort surveys or more local/regional consumption studies. The growing interest in such consumption approaches will stimulate more investigations in this domain.

#### Limitations

The parameters to use are still under debate and need further testing. The present availability of data can be restricted to a limited number of studies, but this figure is expected to markedly improve in the next future.

### A9. Organic/Eco-friendly Production and Consumption

#### Definition

Nowadays, most agro-food productions based on agro-ecological principles are called as “organic” and are certified and labeled at national and continental levels (73).

These well-characterized, controlled, and certified methods of food productions exclude the use of chemical fertilizers, pesticides, GMOs, and intensive animal husbandry (74–79). These methods are acknowledged to better protect environment, biodiversity, and potentially, health consumers.

#### Methodology

Parameters considered are as follows:

– the percentage of consumers buying organic foods and the frequency of consumption.
– organic food consumption in percentage of total food amount or money per capita (e.g., Bionutrinet Cohort Survey in France^7^).
– the percentage of the organic market volume.
– the percentage of land use under organic certification.

#### Data Sources

In most industrialized countries, data on the organic market volume as well as the market shares are available as well as recorded (73). Detailed data for specific food types can be available too.

During some consumer cohort surveys or in national consumption surveys, individual data are collected on organic food consumption (e.g., Germany, France).

Furthermore, national yearly data are now available and continuously recorded (73) regarding the importance of organic food production (number of farms, acreage, volume of foods produced) and share of total. In some countries, agricultural production data (at local/regional/national) are also available along with organic food import/export data (73).

#### Limitations of the Indicator

In some countries, organic production can be marginal only or data on organic production or consumption are not available at national or regional level. But, the availability of data has been and will be increasing (73).

### A10. Physical Activity/Physical Inactivity Prevalence

#### Definition

As physical activity is a key determinant of energy expenditure, it is fundamental to energy balance and weight control. Although there are doubts on considering it as a nutritional indicator or a cofactor of nutritional status, the Working Group decides to consider it in the list of nutritional indicators of sustainability. Anyway, it is important to underline that MD, and more in general, diet needs to be considered a lifestyle, and the regular practice of physical activity is a key component of it.

Physical activity is defined as any bodily movement produced by skeletal muscles that require energy expenditure. The term “physical activity” should not be mistaken with “exercise.” Exercise, is a subcategory of physical activity that is planned, structured, repetitive, and purposeful in the sense that the improvement or maintenance of one or more components of physical fitness is the objective. Physical activity includes exercise as well as other activities, which involve bodily movement and are done as part of working, active transportation, house chores, and recreational activities (80).

Several physical activity indicators have been proposed (81). On the basis of available data, the physical inactivity prevalence has been selected as an indicator of physical activity, using the definition of not meeting any of the following criteria: at least 30 min of moderate-intensity activity per day on at least 5 days per week, or at least 20 min of vigorous-intensity activity per day on at least 3 days per week, or an equivalent combination (82).

#### Methodology

Parameters considered are as follows:

– Attributable disability-adjusted life years (DALYs) from physical inactivity;
– Physical Activity Questionnaires [e.g., WHO Global Physical Activity Questionnaire (GPAQ); International Physical Activity Questionnaire (IPAQ), etc.].

#### Data Sources

National surveys and WHO Global Infobase.

#### Limitations of Indicator

It is difficult to use questionnaires across that are comparable across cultures. All the questionnaires dealing with physical activity presents some limitations in particular, considering the shorter-forms and the versions to be used without personal interview (83). Moreover, data on population-based physical inactivity may be limited in some countries. As an indicator should be, by the way, “monitorable,” it should be based on the data that are readily available, but most of the available data may be difficult to interpret due to differences in the way physical inactivity is measured.

The use of objective methods, such as pedometers, is becoming more feasible, especially with the use of mobile technology and apps that provide such information. However, at the moment, for surveillance activity, the objective methods were rarely used, and there are not available data. Moreover, pedometer is specifically designed to assess walking only; it is enabled to record non-locomotor movements and to examine the rate or intensity of movement. On the other hand, accelerometer is suitable for all populations and is an objective indicator of body movement (acceleration) but is an inaccurate assessment of a large range of activities, and the financial cost may prohibit assessment of large numbers of participants.

Finally, considering the cost–benefit ratio, the Working Group suggests to promote the collection of using harmonized methodologies, such as the same physical activity questionnaire, in all countries (i.e., GPAQ) supported by pedometer or accelerometer.

### A11. Adherence to the Mediterranean Dietary Pattern

#### Definition

Adherence to the traditional MD, or, to diets that resemble the Mediterranean pattern, have been expressed through indexes or scores, defined *a priori*, which operate by combining conceptually and computationally the dietary components that capture the essence of this dietary pattern.

#### Methodology

Parameter considered is as follows:

– Mediterranean Diet Score (MDS) (it ranges from 0 – minimal adherence to the traditional MD – to 9 – maximal adherence):
  - for the five components, which are representative of the MD and are presumed to be consumed in large quantities (vegetables, legumes, fruits and nuts, cereal, and fish), a value of 1 is assigned to persons whose consumption is at or above the components’ sex-specific medians based on the considered sample, and 0 otherwise;
  - a sixth component of the score is the ratio of monounsaturated lipids to saturated lipids, reflecting the principal role of olive oil consumption in the traditional MD: a value of 1 is assigned to persons whose consumption is at or above the sample-specific median of this lipid ratio and 0 otherwise;
  - for the following two components that are presumed to be consumed in low/moderate quantities in the MD (all meats and all dairy products, which are rarely non-fat or low-fat in Mediterranean countries), persons whose consumption is below the median are granted with a value of 1, and persons whose consumption is at or above the median are penalized with a value of 0;
  - for alcohol, a value of 1 is assigned to men who consume between 10 and 50 g of ethanol per day and to women who consume between 5 and 25 g per day, expressing the moderate ethanol consumption in the MD.

#### Data Sources

The MD indexes were estimated in their majority from information collected through detailed Food Frequency Questionnaires (FFQ) or repeated measures of 24-h recall dietary questionnaires, which are not easy to be dealt with, especially from the general public.

#### Limitations

The previous-indicated approach has been very valuable in order to express the whole of a dietary pattern, and specifically of MD. The limitation of the approach is that usually cutoff points used in most scores are sample-dependent, making the interpretation of any identified association of this pattern with health outcomes difficult to generalize. Second, since many MD indexes exist, a natural question is whether some work better than others with respect to capturing the adherence to MD, as well as, to identifying associations of this diet with a specific health outcome. However, to decide which of these numerous MD indexes is “optimal” is rather difficult, since such a decision would require one to evaluate the predictive ability of the various indexes with respect to different outcomes using one population, and then validate the results to different populations. The issue becomes even more complicated due to the population-specific and not universal cutoff values that have been used for discriminating the low/high consumptions for each of the MD components, as described previously. Notwithstanding the scientific value of such an approach, any “optimal” MD scale should also be characterized by its simplicity in the construction of the index as well as in the use of this index widely in public health as well as in clinical practice. Such an investigation would be very important for future studies, which wish to assess the association of MD with health.

### A12. Diet-Related Morbidity/Mortality Statistics

#### Definition

This indicator monitors mortality and morbidity (occurrence of cardiovascular events, type II diabetes, dyslipidemia, hypertension, osteoporosis, neurodegenerative diseases, and some types of cancer) as a proxy for the consumption of healthy diets.

#### Methodology

Parameters considered are as follows:

– prevalence of individuals having physician-diagnosed obesity, cardiovascular diseases (CHD, stroke, and hypertension), type II diabetes, osteoporosis, neurodegenerative diseases, and obesity-related cancers.
– disability-adjusted life year as a measure of overall disease burden expressed of years lost due to illness, disability or early death associated with nutrition-related factors: high blood pressure, high cholesterol (total and LDL), high blood sugar (insulin resistance and/or diabetes).

#### Data Sources

National surveys and WHO World Health Statistics (84, 85).

#### Limitations of the Indicator

Some pathologies can be undiagnosed or underreported in some countries. Data may not be available for the same age groups. If data are not available, mortality prevalence will be used.

### A13. Nutritional Anthropometry

#### Definition

This indicator is based on the body mass index (BMI) and the waist circumference (WC), which are used in a wide variety of contexts as simple methods to assess how much an individual’s body weight departs from what is normal or desirable for a person of his or her height (86).

Body mass index in both men and women represents a measure of underweight (<18.5 kg/m^2^) or different levels of overweight (25–29.9, 30–34.9, 35–39.9, ≥40 kg/m^2^).

Waist circumference in both men and women represents a measure of visceral adiposity (>88 cm in women and 102 cm in men). Increased WC can be a marker for increased risk, even in persons of normal weight associated with insulin resistance.

Overnutrition and undernutrition frequently coexist. Weight loss, real to ideal weight ratio, and specific nutritional assessment tools (Mini Nutritional Assessment – MNA, Just a Nutritional Screening – JANUS) (87, 88) may be useful to detect the presence of malnutrition.

#### Methodology

Parameters considered are as follows:

– undernutrition: prevalence of individuals having a BMI <18.5 kg/m^2^ calculated from self-reported weight and height;
– overweight or obesity: prevalence of individuals having a BMI ≥25.0 kg/m^2^ calculated from self-reported weight and height and/or WC >88 cm in women and 102 cm in men.

Classification of overweight and obesity by BMI, waist circumference, and associated disease risks.

**Table:** 

Disease risk^a^ relative to normal weight and waist circumference				

	BMI (kg/m^2^)	Obesity class	Men 102 cm or less Women 88 cm or less	Men >102 cm Women >88 cm
Underweight	<18.5		–	–
Normal	18.5–24.9		–	–
Overweight	25.0–29.9		Increased	High
Obesity	30.0–34.9	I	High	Very high
	35.0–39.9	II	Very high	Very high
Extreme obesity	40.0+	III	Extremely high	Extremely high

*^a^Disease risk for type 2 diabetes, hypertension, and CVD (http://www.nhlbi.nih.gov/health/public/heart/obesity/lose_wt/bmi_dis.htm)*.

#### Data Sources

WHO Global Database and data locally available through National surveys (89–93).

#### Limitations of Indicator

Individuals tend to overestimate their height and underestimate their weight, leading to underestimation of BMI and of the prevalence of overweight and obesity. Moreover, anthropometric measurements have to be performed by skilled personnel according to a standardized procedure.

Self-reported national surveys might be subject to systematic error (lower reported weight and higher reported height) resulting from non-coverage (e.g., lower telephone coverage among populations of low socioeconomic status), non-response (e.g., refusal to participate in the survey or to answer specific questions), or measurement (e.g., social desirability or recall bias). Data could not be available for some countries.

It is hoped that data of BMI less than 18.5 kg/m^2^ will be available soon from the WHO to measure the rates of undernutrition in these populations.

## Discussion

This paper has identified and summarized some of the most relevant nutritional indicators to measure the sustainability of food consumption. The purpose is, together with additional indicators for the other three sustainability dimensions (environment, economic, and sociocultural), to formulate recommendations for cross-sectoral policy instruments, allowing the comparability and improvement of the sustainability of the diets and food systems in different countries. The selection and analysis of each indicator has been done (wherever possible) with specific reference to the MD as a case study. The sustainability of food systems represents an urgent action area for governments and international organizations to tackle the serious socioeconomic and environmental consequences of short-sighted behaviors and practices involving agricultural land and rural communities (94). It requires developing a set of comprehensive, coherent, integrated, and holistic policies that simultaneously consider the relative priorities (and trade-offs) between different sectors: nutrition, health, lifestyle, society, culture, economy, environment, and agro-biodiversity. The proposed nutrition indicators outlined in this paper will be useful for further developing a methodological framework for designing policies in order, not only to conserve and preserve the traditional diet, such as the MD, as a common cultural heritage and lifestyle but also to enhance the sustainability of dietary models. The MD, in its various national forms, may be used as a case study: a model to describe, understand, and improve the sustainability of current food consumption because of the high and increasing pressure on its fragile natural resources exacerbated by the changes of Mediterranean food consumption patterns (15, 95).

A medium term research and action framework needs to be implemented to analyze the sustainability of the diets in the Mediterranean area (40, 45). The use of the selected indicators and their validation may represent a first step of a “pilot sustainability laboratory” aimed at the definition of a validated procedure that will help governments and policy makers to formulate sustainability-sensitive policies in the promotion of sustainable food systems development in different areas.

The Mediterranean area can be considered as a case study because of its passage through a “nutritional transition” in which problems of undernutrition coexist with overweight, obesity, and food-related chronic diseases (37). Undernutrition is still significant in the South of the Mediterranean: 9.2 million people in 2001–03, 3.9% of the population of the zone, compared with 7.3 million people in 1990–92, 3.8% of the population (96). The rate of stunting among children less than 5 years of age is also very high in many countries in the South: 18% in Algeria, 21% in Egypt, 12% in Lebanon, 24% in Morocco, 12% in Tunisia, and 16% in Turkey. At the same time, according to WHO, overweight and obesity rates in Mediterranean countries continue to rise. Currently reported rates for overweight and obesity range, respectively, from 45.5 and 16.0% in Algeria to 67.9 and 33.1% in Egypt (51).

The indicators that were selected can be attributed to five domains related to nutritional aspects of the diet: *biochemical quality of food* (A3. Vegetable/animal protein consumption ratios; A4. Average dietary energy adequacy; A6. Dietary Energy Density Score; A7. Nutrient density of diet); *food quality* (A2. Fruit and vegetable consumption/intakes; A5. Dietary Diversity Score); *environment* (A8. Food biodiversity composition and consumption; A12. Rate of Local/regional foods and seasonality; A13. Rate of eco-friendly food production and/or consumption); *lifestyle* (A10. Physical activity/Physical inactivity prevalence; A11. Adherence to the Mediterranean dietary pattern), and *clinical aspects* (A1. Diet-related morbidity/mortality statistics; A9. Nutritional Anthropometry). The choice of the indicators is indeed a compromise between what is desirable and what is practical and available in which countries. In one sense, these indicators represent an “ideal” list; it remains to be seen how useful they are for practical application.

In this paper, we refer to databases: FAOSTAT, HBS, EUROSTAT, etc. The quality of the data can be highly variable, and this may represent a limit of the procedure. Anyhow, there is no means to avoid these difficulties, and the presence of different databases may minimize, at least partly, the effects of the deficiencies of each single database.

Thus, the next phase of this work will be:

– Collecting data sets from individual countries to ascertain what figures are available for analysis.
– The validation of the indicators that were selected by the expert group. Thus these indicators will be performed versus sustainability or versus an outcome variable that can be considered a proxy of it.
– The definition of a mathematical model that will be able to combine all the indicators belonging to a single area (e.g., nutritional indicator). In this phase, it will be necessary to verify that all the indicators add some new information to the model, to attribute to each indicator a weight, to avoid collinearity (when a variable can be linearly predicted from the others, it has to be omitted respecting the *lex parsimoniae*).
– Following these steps, we might consider organizing the groups of indicators into a composite index to quantify and monitor sustainability over time. The methodology has been established as a two stage approach to determine first, within each dimension, the relative weightings of the indicators selected and then the weightings between each dimension to enable building a composite index, which may be easily disaggregated into the four dimensions of sustainable diets (45, 97). This methodological approach is sufficiently flexible to allow modifying the type and number of indicators in each dimension as new data accrue.

### Adherence

Recent surveys show that many countries in the Mediterranean area are drifting away from the traditional MD healthy pattern, and current food consumption habits show a decline in their adherence to the MD (26, 31–34, 36). Because of such waning in adherence to the MD, there are major concerns, including health risks [due to an increase in the consumption of lipids (e.g., meat, dairy products, etc.), an increase in the consumption of processed foods, simple carbohydrates (e.g., beverages and foodstuffs with a high carbohydrate content), and a decrease in the consumption of complex carbohydrates (e.g., cereals and legumes) leading to chronic nutrition-related diseases, disability, and increased mortality]; environmental issues (due to an exacerbate ecological footprint as a consequence of a more prevalent consumption of foods from animal sources), and loss of biodiversity (due to the globalization of food production/consumption and the homogenization of eating patterns; the dietary diversity is linked to nutrient composition diversity between foods and among varieties/cultivars/breeds of the same food; dietary diversity may guarantee healthy diet through an adequate presence of nutrients and bioactive molecules) (98). Also, in a time of abundance, larger portion sizes in terms of energy, nutrient, and diversity lead to unhealthy diets, diabesity, and diet-associated morbidity/mortality. Studies on these trends in the Mediterranean area can be useful in promoting more efficacious interventions to preserve healthy eating patterns, to reduce the environmental impact of food production, and to conserve biodiversity.

## Conclusion

The Working Group has selected thirteen indicators from a broader framework of indicators. In this wider group were listed some other indicators, such as food composition, frugality, household food security, level of food processing, nutrient profile, global nutritional index, food losses, and waste, that could be considered a proxy of those selected or overlapping, at least in part, with them. The Working Group will attempt to consider them in the future.

## Author Contributions

All authors listed have made substantial, direct, and intellectual contribution to the work and approved it for publication.

## Conflict of Interest Statement

The authors declare that the research was conducted in the absence of any commercial or financial relationships that could be construed as a potential conflict of interest.

## Footnotes

^1^http://faostat3.fao.org/browse/FB/*/E

^2^http://gesis.org

^3^http://www.nut.uoa.gr/dafneENG.html

^4^http://www.european-nutrition.org/images/uploads/pub-pdfs/European_Nutrition_and_Health_Report_2009.pdf

^5^http://ec.europa.eu/eurostat/web/main/home

^6^e.g., http://www.fao.org/nutrition/education/food-based-dietary-guidelines/regions/countries/finland/en/

^7^http://bionutrinet.etude-nutrinet-sante.fr

## Abbreviations

ADER, average dietary energy requirement; BMI, body mass index; CIHEAM, Centre International de Hautes Etudes Agronomiques Méditerranéennes; CIISCAM, International Inter-University Studies Centre on Mediterranean food culture; DALY, disability-adjusted life years; DDS, Dietary Diversity Score; DES, dietary energy supply; DGA, dietary guidelines for Americans; DVS, Dietary Variety Score; EUROSTAT, statistical office of the European Union; FAO, Food and Agriculture Organization of the United Nations; FBDG, food-based dietary guidelines; FBS, food balance sheets; FFQ, Food Frequency Questionnaire; GHG, green house gas; GPAQ, Global Physical Activity Questionnaire; HBS, Household Budget Surveys; HDDS, Dietary Diversity Score at the household level; IDDS, Individual Dietary Diversity Score; IDS, Individual Dietary Surveys; IFMED, International Foundation of Mediterranean diet; INRAN, Italian National Institute of Food and Nutrition; IPAQ, International Physical Activity Questionnaire; JaNUS, just a nutritional screening; LIM, nutrients to be LIMited; MAR, mean adequacy ratio; MDS, Mediterranean Diet Score; MED, Mediterranean diet; MER, mean excess ratio; MNA, mini nutritional assessment; NDS, Nutrient Density Score; SAIN, Score d’Adéquation Individuel aux recommandations Nutritionnelles; UNESCO, United Nations Educational, Scientific and Cultural Organization; WC, waist circumference; WHO, World Health Organization.
